# Association of Phosphatidylinositol-Specific Phospholipase C with Calcium-Induced Biomineralization in the Coccolithophore *Emiliania huxleyi*

**DOI:** 10.3390/microorganisms8091389

**Published:** 2020-09-10

**Authors:** Onyou Nam, Iwane Suzuki, Yoshihiro Shiraiwa, EonSeon Jin

**Affiliations:** 1 Department of Life Science, Research Institute for Natural Sciences, Hanyang University, Seoul 04763, Korea; oynam@hanyang.ac.kr; 2 Faculty of Life and Environmental Sciences, University of Tsukuba, Tsukuba, Ibaraki 305-8572, Japan; iwanes6803@biol.tsukuba.ac.jp (I.S.); emilhux@biol.tsukuba.ac.jp (Y.S.)

**Keywords:** calcium, biomineralization, coccolith, *Emiliania huxleyi*, phosphatidylinositol-specific phospholipase C

## Abstract

Biomineralization by calcifying microalgae is a precisely controlled intracellular calcification process that produces delicate calcite scales (or coccoliths) in the coccolithophore *Emiliania huxleyi* (Haptophycea). Despite its importance in biogeochemical cycles and the marine environment globally, the underlying molecular mechanism of intracellular coccolith formation, which requires calcium, bicarbonate, and coccolith-polysaccharides, remains unclear. In *E. huxleyi* CCMP 371, we demonstrated that reducing the calcium concentration from 10 (ambient seawater) to 0.1 mM strongly restricted coccolith production, which was then recovered by adding 10 mM calcium, irrespective of inorganic phosphate conditions, indicating that coccolith production could be finely controlled by the calcium supply. Using this strain, we investigated the expression of differentially expressed genes (DEGs) to observe the cellular events induced by changes in calcium concentrations. Intriguingly, DEG analysis revealed that the phosphatidylinositol-specific phospholipase C (PI-PLC) gene was upregulated and coccolith production by cells was blocked by the PI-PLC inhibitor U73122 under conditions closely associated with calcium-induced calcification. These findings imply that PI-PLC plays an important role in the biomineralization process of the coccolithophore *E. huxleyi*.

## 1. Introduction

Coccolithophores play an essential role in the global carbon cycle through considerable carbon dioxide fixation and carbon sequestration as they are major producers of marine biogenic calcites and produce large blooms in the open ocean. *Emiliania huxleyi* (*E. huxleyi*), a calcifying haptophyte, is widespread throughout the oceans worldwide [1,2], including polar [3] and coastal regions [4]. Due to their ability to form dense blooms at mid and high latitudes, they are intensively studied to understand the environmental implications of calcification [5,6]. Furthermore, they produce ornate calcium carbonate scales, known as coccoliths, as cell covering. The calcite plate originates from a separate intracellular compartment known as the coccolith vesicle (CV) [7,8,9,10]. Moreover, since the complex shape of calcite crystals, which is similar to coccoliths, cannot be produced artificially at a nanometer scale, coccoliths are an attractive biogenic product for bioengineers and material scientists and are desirable for various applications in nanotechnology [11]. Despite the pivotal roles of this calcifying alga, the underlying molecular mechanism remains unknown. Understanding the critical molecular mechanism underlying this complicated biological mineralization process will not only provide a profound insight into the biological function but also advance our knowledge of such processes, thus facilitating mimicry of this complex biogenic morphology.

Pioneering studies by various research groups attempted to elucidate the mineralization process in the bloom-forming species *E. huxleyi*. Thus far, various factors affecting biomineralization, such as nutrient limitation, life cycle, temperature, and ocean acidification, have been identified [8,12,13,14,15,16]; several studies using the non-calcifying *E. huxleyi* strain CCMP 1516 (synonym: CCMP 2090), which lacks coccolith production, identified calcification-related genes under phosphate-limited conditions, which are usually known to increase calcification [17,18,19,20]. These studies examined the differential expression of biomineralization-related genes in calcifying and non-calcifying cells under phosphate-limited and -replete conditions, respectively. Furthermore, gene expression in haploid (non-calcifying) and diploid (calcifying) cells was analyzed in *E. huxleyi* strains RCC 1217 (1N) and RCC 1216 (2N), respectively [21,22].

Meanwhile, *E. huxleyi* cells acclimated to different calcium concentrations ([Ca^2+^]) were examined to understand calcification-related gene regulation [22,23,24]. In our previous study (Nam et al., 2019), *E. huxleyi* CCMP 371 cells were grown for 20 generations to adjust their physiological states to three different [Ca^2+^] conditions; then, these cells were used to identify genes whose expression was strongly regulated by an association with the intracellular calcification process. Notably, phosphatidylinositol-specific phospholipase C (PI-PLC) was enriched in the gene ontology (GO) terms in calcifying cells grown at 10 mM [Ca^2+^] in comparison with non-calcifying cells grown at 0 mM [Ca^2+^] [24].

In the literature, PI-PLC was shown to cleave the polar phosphate head of phospholipids to produce diacylglycerol and inositol triphosphate, which are then released into the cytoplasm [25]. In mammalian cells, PI-PLC is involved in vesicle transport, cytoskeletal reorganization, intracellular signal transduction, ion channel function, endocytosis, exocytosis, mitosis, and neuronal signal transduction [26]. Based on this information, PI-PLC may be a possible candidate closely associated with coccolith production, since coccolith formation in CV is regulated by a precise endomembrane system. However, the contribution of PI-PLC in the intracellular coccolith formation process of *E. huxleyi* has yet to be demonstrated experimentally.

In this study, we designed experiments to clearly reveal the effect of [Ca^2+^] enrichment by the sudden transfer of cells grown in low (0.1 mM) [Ca^2+^] to ambient (10 mM) [Ca^2+^] conditions on gene expression, including the *PI-PLC* gene, and coccolith production in *E. huxleyi* CCMP 371. This design differed from that of our previous study [24] and was very useful for observing time-dependent changes in cellular processes that are closely associated with calcium-induced coccolith production. First, we demonstrated that coccolith production by this strain is directly regulated by [Ca^2+^] concentrations and therefore calcium-induced biomineralization but is not affected by phosphate-limitation stress, which is known to be an important calcification-promoting factor in the other strains of *E. huxleyi*. Second, after transferring cells grown in 0.1 mM [Ca^2+^] (non-calcified cells) to 10 mM [Ca^2+^] to stimulate coccolith production, transcriptome analysis was performed to identify differentially expressed genes (DEGs) that are strongly related to calcium-induced biomineralization. Domain enrichment analysis of upregulated DEGs revealed that PI-PLC is associated with calcium-induced biomineralization and plays a vital role in signal transduction as analyzed by the protein–protein interaction network. Third, the PI-PLC inhibitor U73122 suppressed coccolith production, suggesting that PI-PLC is associated with the calcium-induced biomineralization process in *E. huxleyi* CCMP 371. This is the first study, to our knowledge, to elucidate the role of PI-PLC in the biomineralization of the coccolithophorid alga *E. huxleyi*. 

## 2. Materials and Methods

### 2.1. Algal Strain and Culture Conditions

*E. huxleyi* strain CCMP 371 was purchased from the Provasoli-Guillard National Center for Marine Algae and Microbiota (East Boothbay, ME, USA); this strain has unique characteristics for calcification as described in the introduction. The cells were cultured in sterile artificial seawater [27] enriched with nitrate, phosphate, trace metals, and vitamins at f/2 concentrations [28] as well as 0.01 μM selenium [29]. The cells were maintained at 20 °C under constant irradiation at 100 μmol m^−2^ s^−1^ and agitated twice daily by manual shaking to homogenize the culture. To inhibit PI-PLC, the cells were treated with U73122 (Sigma-Aldrich Co., St. Louis, MO, USA) at final concentrations of 0.01, 0.1, and 0.2 μM. U73122 is a PI-PLC and A_2_ inhibitor that inhibits the hydrolysis of phosphatidylinositol to inositol triphosphate, leading to a decrease in free cytosolic Ca^2+^; it also acts to inhibit the coupling of G-protein phospholipase C activation while being unaffected by cAMP production (U6756, Product Information Sheet, Sigma). As a negative control, U73343 (Sigma-Aldrich Co., St. Louis, MO, USA), a less active structural analog of U73122, has been used for the nonspecific effects of U73122 on PI-PLC. As U73122 and 73343 were dissolved in dimethyl sulfoxide (DMSO), control cells were treated with DMSO only. Twenty-four hours after inoculating the cells, U73122, U73343 and DMSO were applied, and [Ca^2+^] was concurrently shifted from 0.1 to 10 mM. Cells were harvested after 72 h of treatment for further experiments using flow cytometry in triplicate. 

### 2.2. Inorganic Phosphate Measurement

The inorganic phosphate (Pi) in the medium was analyzed by the molybdenum blue method [30]. For the analysis, 20 μL of acid ascorbate solution (2.5% ammonium molybdate, 0.1% potassium antimonyl tartrate sesquihydrate, and 3.15 M H_2_SO_4_), and 20 μL of acid molybdate solution (10% ascorbic acid and 2.25 M H_2_SO_4_) was added to 1 mL of the supernatant of the cell suspension. 

### 2.3. AP Activity Measurement

According to the method described by Reichardt et al. [31], alkaline phosphatase (AP) activity was estimated using *p*-nitrophenyl phosphate (*p*-NPP) as a substrate to measure its degradation to *p*-nitrophenol. First, *p*-NPP (100 μL, 36 mM) and 700 μL of 3-(cyclohexylamino)-1-propane sulfonic acid buffer (200 mM, pH 10.0) was added to 200 μL of the cell suspension with intact cells, followed by incubation at 40 °C for 15 min. The reaction was terminated by adding 100 μL of 4 M NaOH, and the absorbance at 397 nm was measured using a UV-VIS spectrophotometer (UV mini 1240; Shimadzu, Kyoto, Japan). 

### 2.4. Determination of TIC and TOC

According to Pelusi et al. [32], 50 mL of each cell culture sample was harvested during the exponential growth phase. Half of the culture sample (25 mL) was centrifuged (1720× *g*, 5 min) to remove cells. Then, the supernatant was filtered using a Millex HA filter (pore size 0.45 μm, Merck Millipore, Darmstadt, Germany) to acquire culture medium without any particles, including cell debris (culture medium fraction). Total organic carbon (TOC) and total inorganic carbon (TIC) values of both the cell suspension and culture medium fraction (25 mL each) were measured using a TOC analyzer (Shimadzu Corporation, Kyoto, Japan). TIC was calculated by subtracting TOC from the total carbon. The cellular organic and inorganic carbon quantities (Cell C and Cell IC, respectively) were calculated using the following equations:

Cell C = (TOC in cell suspension) − (TOC in culture medium fraction),(1)

Cell IC = (TIC in cell suspension) − (TIC in culture medium fraction),(2)

where the TOC and TIC values are expressed as cells contained in a 1-L culture (mg/L). 

### 2.5. Cell Growth and Microscopic Observation

The cell number was determined by counting the cells using a hemocytometer (Marienfeld, Bad Mergentheim, Germany) or a cell counter (Sysmex CDA-1000, Kobe, Japan). Cells surrounded by coccolith were observed under polarized light, and micrographs were taken using a microscope (BX53; Olympus Ltd., Tokyo, Japan) equipped with a fluorescent microscope digital camera (Keyence, Osaka, Japan). 

### 2.6. Sample Preparation

Triplicate samples from each time point (−3, 0, 1, and 3 h before and after loading to 10 mM [Ca^2+^]) were harvested in a conical tube by centrifugation (1720× *g*, 5 min, 4 °C) and immediately frozen in liquid nitrogen. The total RNA was isolated for RNA-seq using TRIzol reagent (Invitrogen, Carlsbad, CA, USA), the RNeasy Mini Kit (Qiagen, Hilden, Germany), and DNase (Qiagen, Hilden, Germany) treatment according to the manufacturer’s instructions. RNA quality and integrity were estimated using an Agilent Technologies 2100 Bioanalyzer (Agilent Technologies, Santa Clara, CA, USA). 

### 2.7. RNA-seq and Transcriptome Analysis

Library construction and RNA-seq using an Illumina HiSeq 3000 (Illumina, Inc., San Diego, CA, USA) were performed by Macrogen (Seoul, Korea). The sequencing libraries were prepared according to the manufacturer’s instructions using the Illumina TruSeq 3000 SBS Kit v3 and sequenced in pair-end reads (Table S1). Read quality was assessed using FastQC v0.11.5 [33]. The raw reads were trimmed with Trimmomatic v0.36 [34]. Trimmed reads for each sample were aligned using Bowtie v1.1.2 (http://bowtie-bio.sourceforge.net/index.shtml) with the previously assembled reference transcripts (DDBJ/EMBL/GenBank accession GHJP00000000) [24]. Sequenced raw reads are deposited in the Sequence Read Archive of the National Center for Biotechnology Information under Bioproject number PRJNA623073. 

The RSEM algorithm (v1.2.29) was used to count the aligned reads, and contigs with 0 counts in all 12 samples were removed from the analysis [35]. Thus, we conducted statistical analysis of 16,061 contigs after excluding 32,589 out of a total of 48,650 contigs. Unigenes were distinguished as DEGs if they showed a fold change (| log_2_FC |) > 1 between the 0.1 mM calcium group (−3 and 0 h (time of the shift), Group 1) and 10 mM calcium group (1 and 3 h, Group 2) using a cut-off probability score to ensure a false discovery rate (FDR) less than 0.05. The domain enrichment (pfam and Interpro) and protein–protein interaction networks of DEGs were analyzed using STRING (v11.0) [36]. The interaction score parameters were set at the medium confidence level for minimum requirements. 

### 2.8. qPCR

Total RNA was extracted from cell cultures using TRIzol reagent (Invitrogen, Carlsbad, CA, USA) and the RNeasy Mini Kit (Qiagen, Hilden, Germany); cDNA was synthesized using a Superscript III Kit (Invitrogen, Carlsbad, CA, USA) primed with oligo dT primers, following the manufacturer’s protocol. The cDNA was used as a template for qPCR. SYBR green was applied for amplicon detection. SYBR premix (Takara, Tokyo, Japan) and the Thermal Cycler Dice Real-Time System TP8200 (Takara, Tokyo, Japan) were used for cDNA amplification. Actin was used as the reference gene for qPCR [22]. Target sequences of primers are listed in Table S2. 

### 2.9. Calcein Staining and Flow Cytometry Analysis

For each sample, 7 mL of cells were used for calcium carbonate staining by calcein (Sigma-Aldrich Co., St. Louis, MO, USA). Cells were calcein labelled for 2 h according to Fox et al. [37] and washed three times by gentle centrifugation (840× *g*, 1 min, 20 °C). Then, cells were resuspended in fresh f/2-si media before flow cytometry analysis. The BD FACS Canto II flow cytometer (BD Biosciences, San Jose, CA, USA) was used to analyze the calcein-stained *E. huxleyi* samples. A 488-nm excitation laser with an FITC emission filter was used to detect calcein fluorescence and with a PerCP-Cy5.5 filter to detect chlorophyll autofluorescence. The populations of cells and coccoliths were distinguished by FSC and side scatter (SSC). In total, of 10,000 or 20,000 cells were gated, and chlorophyll-positive populations were counted for each sample. Data were analyzed using FlowJo software version 10.0.7 (Tree Star, Ashland, OR, USA). Statistically significant differences were determined by Student’s *t*-test (* *p* < 0.05, ** *p* < 0.01).

## 3. Results

### 3.1. Coccolith Formation in E. huxleyi CCMP 371 under Phosphate-Replete Conditions

**Figure 1:**
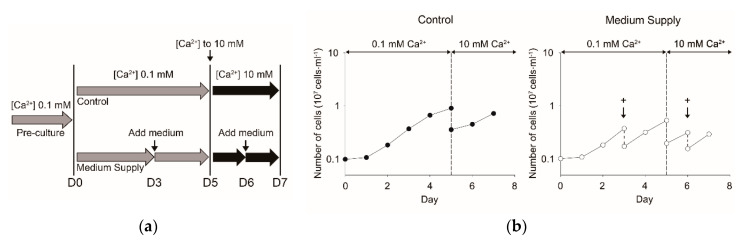

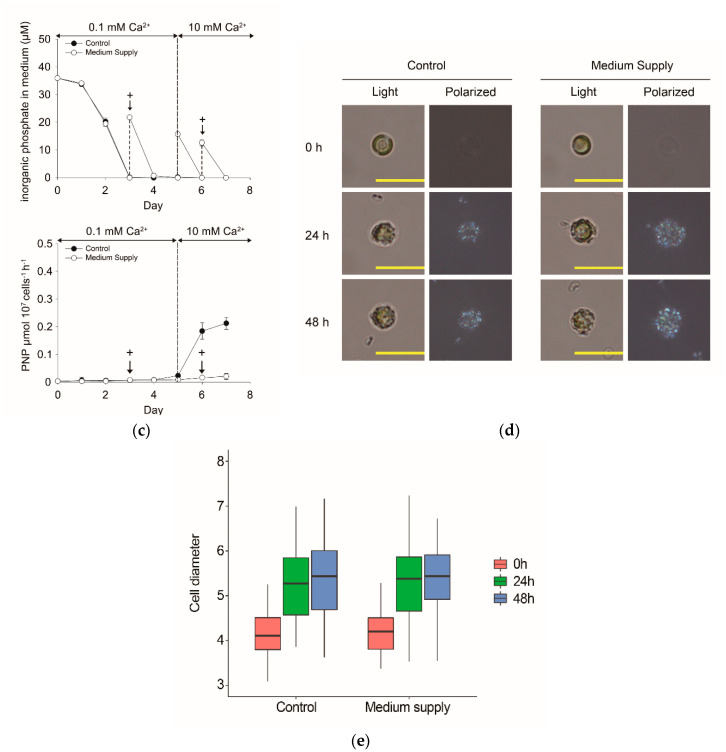
Changes in the physiological and morphological data of coccolith production by the coccolithophore *E. huxleyi* CCMP 371 when cells grown in low [Ca^2+^] were suddenly transferred to ambient [Ca^2+^] conditions under Pi-replete and -limited conditions. (**a**) Experimental design showing the manner in which [Ca^2+^] and [Pi] were controlled during this experiment. Cells grown in low [Ca^2+^] (pre-culture) were transferred into the same Ca^2+^-limited (0.1 mM) medium in control and medium supply (MS) conditions on Day 0. On Day 3, the fresh Ca^2+^-limited medium was added to the MS culture (indicated by arrows [with +]) but not the control. On Day 5, both control and MS cultures were suddenly transferred to 10 mM [Ca^2+^] conditions. Thereafter, the MS culture was diluted once more with the ambient seawater medium on Day 6; the control was not diluted. (**b**) Change in growth as monitored by cell number. (**c**) Changes in [Pi] in the culture medium and external alkaline phosphatase activity (expressed as PNP) as a parameter of Pi limitation. (**d**) Microscopic observation under light and polarized microscopes for monitoring whole-cell images and coccoliths produced as cell covering, respectively. The cell image presented is one typical example. The yellow bar indicates a 10-μm scale. (**e**) Cell diameter measured and shown as boxplots. More than 60 individual cells were examined, and the box color indicates the time point after shifting [Ca^2+^] from 0.1 to 10 mM (red: 0 h, green: 24 h, blue: 48 h, cell diameter: μm).

Firstly, cells cultivated in ambient seawater culture medium containing 10 mM calcium chloride and 36 μM inorganic phosphate (Pi) as the stock culture were transferred to culture medium containing 0.1 mM [Ca^2+^]. Then, the cells grown in 0.1 mM [Ca^2+^] (pre-culture) were transferred into two culture vessels containing the fresh culture medium with 0.1 mM [Ca^2+^] at Day 0. The vessels were maintained at 0.1 mM [Ca^2+^] until Day 5, when Ca^2+^ solution was added to attain 10 mM [Ca^2+^]; subsequently, they were maintained until Day 7. In one culture vessel, [Pi] linearly decreased to zero on Day 3, and Pi-depleted conditions were maintained until Day 5 (Control in Figure 1a). In the other vessel, the cell suspension was diluted with fresh medium on Days 3 and 6 to avoid Pi-depleted conditions (medium supply (MS) in Figure 1a). Both cultures remained in the exponential growth phase during the experiment (Figure 1b), even under different Pi conditions (Figure 1c). The change in extracellular alkaline phosphatase (AP) activity, induced by Pi limitation in the cells, revealed that Pi limitation started on Day 5 in the control but was not observed in MS conditions (Figure 1c). AP activity expressed on the cell surface was determined as a parameter of Pi limitation since the activity is known to be induced quantitatively depending on the extent of Pi limitation in *E. huxleyi* [13]. Cells grown in 0.1 mM [Ca^2+^] and harvested on Day 5 (0 h in Figure 1d) had no coccolith on the cell surface in both control and MS conditions. Coccolith production started already at 24 (Day 6) and 48 h (Day 7) after the shift to ambient [Ca^2+^] (10 mM), and the amount of coccoliths covering the cells became more obvious in MS than in the control (Figure 1d,e). The results indicate that coccolith production in *E. huxleyi* CCMP 371 is mainly regulated by [Ca^2+^] conditions, but not Pi concentration.

### 3.2. Morphological and Quantitative Monitoring of Calcium-Induced Coccolith Production

**Figure 2:**
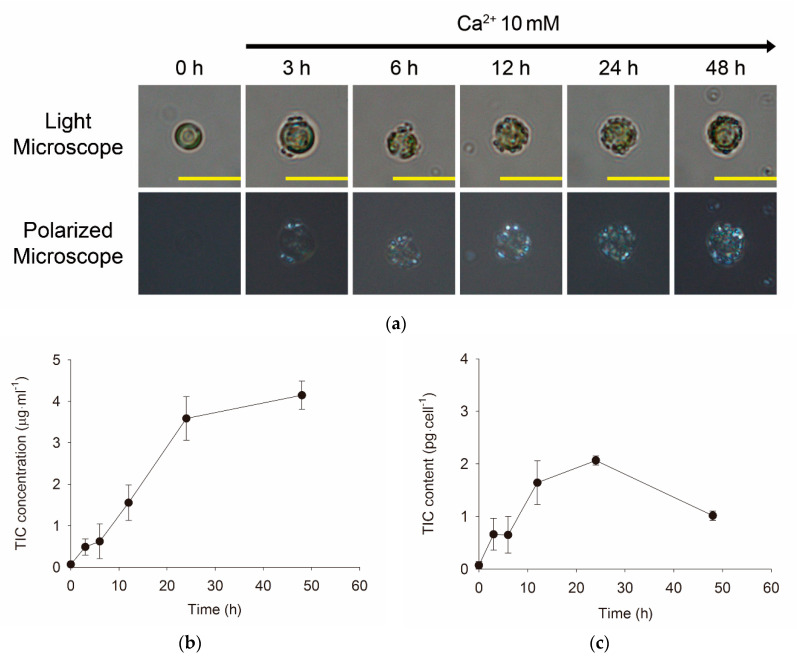
Rebuilding of the coccosphere and TIC of *E. huxleyi* CCMP 371 cells after transferring cells grown in low [Ca^2+^] to the ambient [Ca^2+^] conditions. (**a**) Whole-cell images under light and polarized microscopes. The yellow bar indicates a 10-μm scale. (**b**) TIC concentration in the cell fraction, namely cells with coccoliths (Cell IC/mL cell fraction). (**c**) TIC content in a cell (Cell IC per cell).

According to the observations under light and polarized microscopes, cells pre-cultivated at 0.1 mM [Ca^2+^] for 5 days (0 h) were confirmed to have no coccolith (Figure 2a). Subsequently, after shifting [Ca^2+^] from 0.1 to 10 mM in MS conditions with sufficient [Pi], coccolith production began to increase the amount of calcite scales surrounding the cells (coccoliths). The increase in the TIC concentration in the cells was directly proportional to the coccolith formation observed by light and polarized microscopes (Figure 2). These data show that coccolith formation was induced by the increase in calcium concentration. Meanwhile, the quantity of coccoliths produced as CaCO_3_ (which was estimated as TIC by a TOC analyzer that quantified both TIC and TOC as described in ‘Materials and Methods’) increased during calcification for 24 h and then decreased slightly toward 48 h in the cell fraction (Figure 2b) and cells (Figure 2c). Both TIC (TIC/mL) and TOC (TOC/mL) in the cell fraction increased linearly as observed with coccoliths in the whole cells, whereas cellular TOC was maintained at nearly constant levels owing to the increase in cell number during the experiment (Figure S1). The decrease in TIC in the medium was quantitatively accompanied by an increase in TIC in the whole cells (Figure S1c), indicating that most carbon absorbed from the medium was transferred into the coccoliths. 

### 3.3. Calcium-Induced Biomineralization-Associated Genes

**Figure 3:**
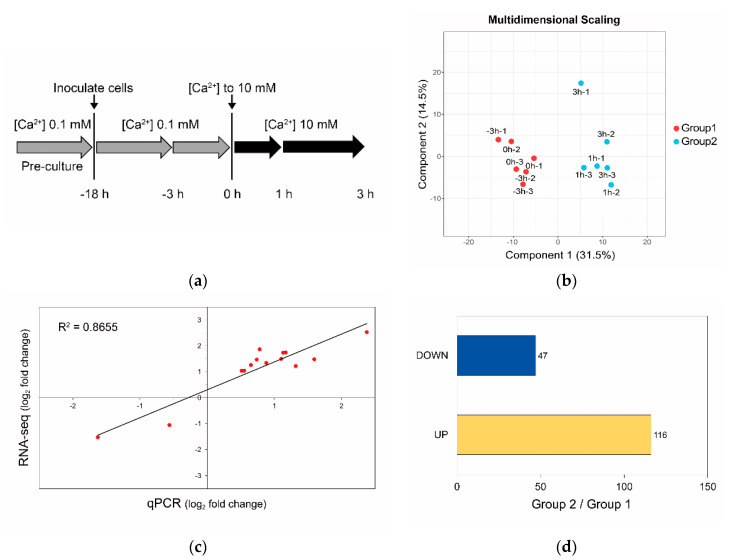
Transcriptomic analysis to identify genes that were upregulated with increasing calcium-induced coccolith production in *E. huxleyi* CCMP 371 cells. (**a**) The overall sampling protocol for RNA-seq uses the same experimental design as MS in Figure 1 and Figure 2. (**b**) The multidimensional scaling plot categorized into two groups depending on sampling time before (−3 and 0 h, red dots) and after adding 10 mM [Ca^2+^] (1 and 3 h, light blue); namely, group 1 involving non-calcifying cells grown at low [Ca^2+^] conditions and group 2 involving Ca^2+^-induced calcified cells, respectively. (**c**) Analysis of differentially expressed genes (DEGs) by qPCR (biological triplicates, *n* = 3) vs. RNA-seq plot. (**d**) The numbers of upregulated (yellow) and downregulated (blue) DEGs in Group 2 compared with Group 1 (Group 2/Group 1).

The calcium-induced calcifying cells were already observed at 3 h after shifting [Ca^2+^] from 0.1 to 10 mM (Figure 2). Therefore, the cells at 1 and 3 h after the shift were used to analyze genes influenced by calcium at the transcriptional level (Figure 3a); additionally, samples harvested at 0 and 3 h before the shift (−3 and 0 h, respectively) were examined. These samples were used for RNA sequencing (RNA-seq) (Figure 3a) by generating an average of 68 million reads per sample (Table S1). Mapping of these sequenced reads against the previously assembled transcripts [24] clearly showed that samples were separated by shift time into groups of DEGs: before (Group 1: non-calcified cells harvested at −3 and 0 h) and after (Group 2: calcified cells harvested at 1 and 3 h) shifting [Ca^2+^] from 0.1 to 10 mM (Figure 3b). Expression levels verified by quantitative polymerase chain reaction (qPCR) with randomly selected DEGs confirmed the differential gene expression between the two groups (Figure 3c, Table S2). Comparison of DEGs between groups revealed that the number of upregulated and downregulated DEGs was 116 and 47, respectively, in cells calcified by [Ca^2+^] enrichment (Group 2) (Figure 3d, Table S3). 

The enriched domain search using public databases identified transcripts encoding phosphoinositide (PI) system and polyketide synthase (PKS) as functioning DEGs that were upregulated with [Ca^2+^]-stimulated coccolith production (Table 1 and Table 2). However, we did not find any domains enriched from the downregulated DEGs. Notably, PI-PLC domains were identified among the PI system in the PFAM database (PF00388 in Table 1) and the Interpro database (IPR000909, IPR001711, and IPR001192 in Table 2). Accordingly, GO enrichment analysis indicated PI-PLC as the gene that was upregulated in cells grown in 10 mM [Ca^2+^] in our previous report [24]. Additionally, according to the protein–protein interaction network of DEGs, which are essential for understanding the cellular processes induced/activated under calcium-replete conditions, these upregulated DEGs showed an important role of PI-PLC in signal transduction (Figure S3).

### 3.4. Inhibition of Coccolith Production by PI-PLC inhibitor U73122

**Figure 4:**
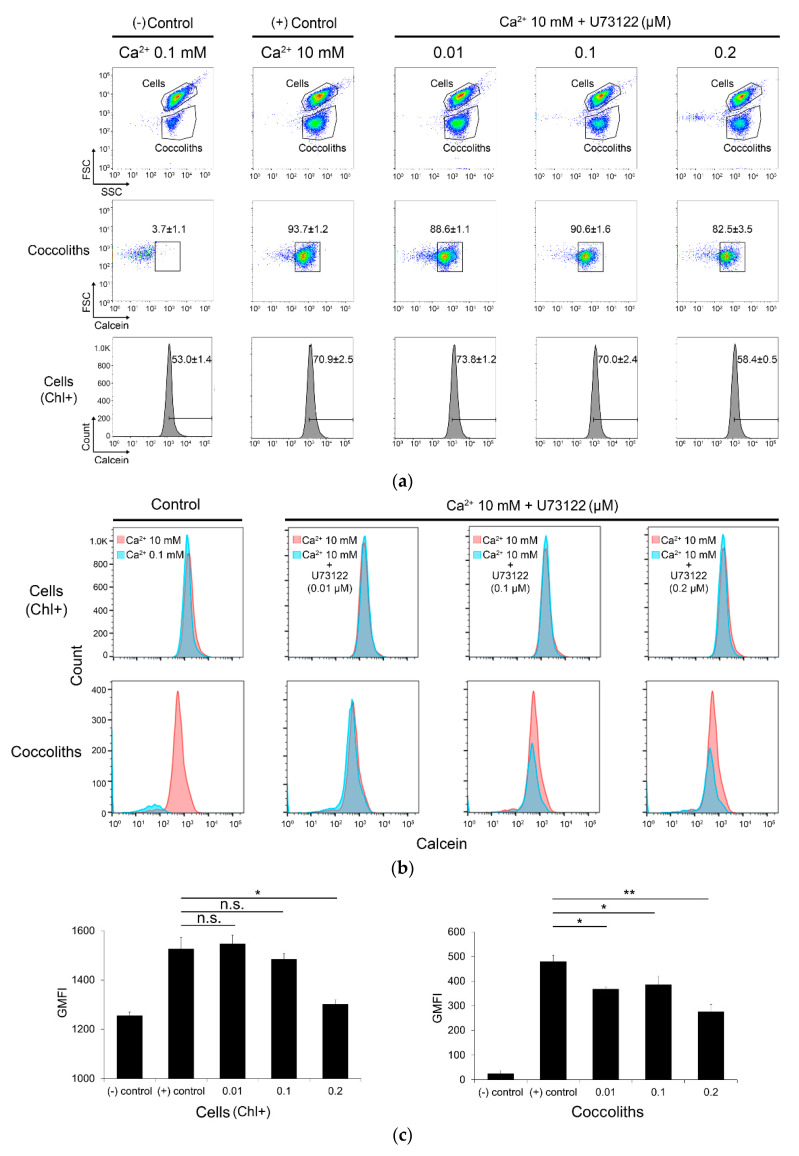
Effect of the PI-PLC inhibitor U73122 on calcium-induced coccolith production analyzed using a flow cytometer in *E. huxleyi* CCMP 371. Cell growth data are provided in Figure S2. To provide samples, we transferred cells grown in 0.1 mM [Ca^2+^] (- Control) to 10 mM [Ca^2+^] (on Day 1 in Figure S2) and incubated them for 72 h without (+ Control) and with 0.01, 0.1, and 0.2 μM U73122 (until Day 4 in Figure S2). (**a**) Data of forward scatter (FSC) vs. side scatter (SSC) (top), and FSC vs. calcein-positive populations estimated from the coccolith-gated population (middle) and cells (chlorophyll-positive, Chl+) (bottom). (**b**) Merged histograms of counts vs. calcein-positive populations in cells (Chl+) (upper) and coccoliths (lower). Boxes on the left represent data for the control without inhibitor (pink: 10 mM [Ca^2+^]-treated cells; blue: cells grown in 0.1 mM [Ca^2+^], boxes on the left). Other boxes represent data for samples treated with 0.01, 0.1, and 0.2 μM U73122 (designated as 10 mM Ca^2+^ + U73122 μM) (blue) and 10 mM [Ca^2+^]-treated cells (pink), as indicated in each box. (**c**) The geometric mean fluorescence intensity (GMFI) of the cells and coccoliths. Statistically significant differences were determined by Student’s *t*-test (* *p* < 0.05, ** *p* < 0.01).

Based on the identification of PI-PLC as a calcium-upregulated gene, the effect of the PI-PLC inhibitor U73122 on coccolith production was tested in *E. huxleyi* CCMP 371. We first examined the effect of U73122 at 0.01, 0.1, and 0.2 μM on cell growth; consequently, no inhibition of cell growth was observed for at least for 3 days when 10 mM [Ca^2+^] and U73122 were simultaneously added to cells grown in 0.1 mM [Ca^2+^] on Day 1 (Figure S2). Based on these results, the effect of U73122 on coccolith production during the culture (as shown in Figure S2) was monitored by flow cytometry using a previously established calcein staining method, in which calcein binds to calcium ions in biominerals such as coccoliths [37]. This method allowed calcium detection in both coccoliths that were attached and those that were detached from the cells as a larger or smaller forward scatter (FSC, cell size), respectively (Figure 4a). Cells were additionally gated as chlorophyll-positive populations. The calcein-positive cell population was analyzed under each condition to estimate the quantity of coccoliths that were attached to intact cells or detached in the medium. The FSC value of the organic parts of the cells (recognized with chlorophyll fluorescence and shown as ‘Cells (Chl+)’ in Figure 4) was suppressed by 0.2 µM U73122 to 17% of the control, whereas that of the coccoliths was greatly diminished to 51% of the control at 10 mM [Ca^2+^] (Figure 4a,b). The average geometric mean fluorescence intensity of the coccolith-gated calcein-positive population showed a significant decrease in all three U73122-treated conditions (23%, 20%, and 42% less at 0.01, 0.1, and 0.2 µM, respectively) (Figure 4c). However, the coccolith production examined by U73343 treatment, which is known as an inactive analog of U73122, did not show a significant decrease in U73343-treated conditions compared to the control (Figure S4). These results suggest the involvement of PI-PLC in the calcium-induced biomineralization by *E. huxleyi* CCMP 371.

## 4. Discussion

### 4.1. Physiological Regulation of Cell Culture for Providing Calcium-Induced Calcified Cells

In this study, we first established a physiologically well-organized cell cultivation system to investigate the calcium-specific induction of intracellular calcification using the coccolithophore *E. huxleyi* CCMP 371 strain. To identify genes closely associated with coccolith production as well as intracellular calcification, we performed transcriptomic analysis in *E. huxleyi* cells by transferring non-calcifying cells grown in low [Ca^2+^] to ambient seawater medium containing 10 mM [Ca^2+^], where coccolith production was greatly stimulated (Figure 1 and Figure 2). To promote coccolith production by cells, we selected 10 mM [Ca^2+^] (final concentration) based on the ambient seawater calcium concentration (10 mM) that is reported to be the optimal condition for calcification in the literature [22,23,24,38,39,40,41].

This study provided experimental evidence supporting the fact that the calcification of *E. huxleyi* CCMP 371 is induced by the calcium supply and is independent of Pi conditions, since coccolith production was observed in both Pi-limited and -replete conditions (Figure 1). Therefore, the *E. huxleyi* CCMP 371 strain is very useful for investigating calcium-induced biomineralization without the effects of Pi. As phosphate limitation is generally known to increase calcification in other strains of *E. huxleyi* [13,18,19,20], the regulatory mechanism appears to differ among strains, even within *E. huxleyi*. This strain-specific variation may be supported by evidence showing a wide variation of *Emiliania* genomes, including pan-genomes among strains isolated from various environments [42].

The quantitative estimation of coccoliths and cellular organic materials as TIC and TOC, respectively, by a TOC analyzer demonstrated that the cellular IC content (cell IC) represents coccoliths produced from IC absorbed from the carbon in the medium (Figure 2 and Figure S1). Although the total TOC in the cell fraction increased along with the cell number, the cellular TOC content (Cell C) remained nearly constant during culture at ambient [Ca^2+^] (Figure S1), indicating that the increase in [Ca^2+^] resulted in the promoted uptake of both calcium and bicarbonate, followed by CaCO_3_ crystallization for coccolith production.

We observed visible coccoliths on the cell surface at 3 h after the [Ca^2+^] shift (Figure 2a). The data are comparable with previous data by Paasche (1962) [43], who reported that the production of one coccolith takes less than 3 h under illumination, although the time taken varies among strains. Therefore, the experimental design used for the transcriptomic analysis of calcium-induced *E. huxleyi* CCMP 371 cells in this study was adequate for understanding the molecular mechanisms of calcium-induced coccolith production as an important biomineralization mechanism.

### 4.2. Identification of Calcium-Induced Biomineralization-Associated Genes

Using the established experimental design, transcriptomic analysis was performed to identify calcium-induced biomineralization-associated genes in *E. huxleyi* CCMP 371 (Figure 3). For RNA-seq in this study, over 50 million reads per sample (in four conditions with three biological replicates) were generated and processed for mapping the reads against previously assembled transcripts by Nam, Park, Lee and Jin [24]. The multidimensional scaling represents the degree of similarity between samples and shows that groups 1 and 2 were distinguished by the [Ca^2+^] shift. The transcriptional response to calcium-induced biomineralization resulted in the identification of 116 upregulated and 47 downregulated DEGs. The enriched domains in the upregulated DEGs were associated with polyketide synthesis, signal transduction, and organelle transport (Table 1 and Table 2). Inversely, no domains were enriched in the downregulated DEGs, probably owing to the small number of gene lists.

### 4.3. Association of PKS with Calcium-Induced Biomineralization

Several findings have linked PKS with calcification in the literature: (1) the biosynthesis of polyketides shares large similarities with fatty acid biosynthesis in bacteria, fungi, and plants [44]. (2) The link between PKS and calcium carbonate biomineralization was reported in teleost fish medaka *Oryzias latipes* [45]. In a spontaneous medaka mutant, PKS facilitated nucleation in otolith (calcareous ear stone) mineralization. (3) During the developmental stage of sea urchins, PKS expression is limited to skeletogenic cells and their precursors, which are responsible for calcium carbonate biomineralization [46]. The following data were found for *E. huxleyi* in the literature: (1) type I PKS genes in *E. huxleyi* were previously reported by John et al. [47]. (2) Microarray analysis revealed high PKS levels in a calcifying diploid strain of *E. huxleyi* RCC 1216 [48]. (3) Proteomic data obtained in *E. huxleyi* confirmed the expression of the modular type I PKS protein [49]. Despite these reports, the association of PKS with biomineralization has rarely been reported in *E. huxleyi*.

In the current study, PKS transcription was upregulated after the shift from 0.1 to 10 mM [Ca^2+^], which might be associated with biomineralization. Actually, many functional domains associated with type I PKSs, such as ketoacyl synthase (PF02801, PF00109, IPR14031, IPR14030, IPR018201), ketoacyl reductase (PF08659), acyl transferase (IPR016035), enoyl reductase (IPR020843), acyl carrier protein (IPR036736), dehydratase (IPR020807), and thioesterase (IPR001031), were identified to be upregulated by the increase in [Ca^2+^] used to induce and stimulate coccolith production (Table 1 and Table 2). These data demonstrate that PKS has a possible role in calcium-induced calcification in *E. huxleyi*. Therefore, unveiling the relationship between PKS and biomineralization may advance the present knowledge on this bloom-forming cosmopolitan coccolithophorid alga *E. huxleyi*.

### 4.4. Association of PI-PLC with Calcium-Induced Biomineralization

Among the enriched domain functions related to signal transduction and organelle transport in DEGs upregulated by the elevation of [Ca^2+^], phosphatidylinositol-specific phospholipase C (PI-PLC) and the phosphoinositide PI-PLC family were clearly identified as possible candidates associated with calcium-induced biomineralization (PF00388, IPR000909, IPR001711, and IPR001192 in Table 1 and Table 2). These results, obtained in the present study, strongly confirm our previous study results, which suggested the possibility that PI-PLC participates in calcification in calcium-enriched *E. huxleyi* cells [24]. Further, the protein–protein interaction network analysis performed in the present study confirmed that the PI system is also working in the coccolith production mechanism in *E. huxleyi* strain CCMP 371 (Figure S3).

Concerning the function of PI-PLC, PI-specific PI-PLC catalyzes the degradation of PIs, known as regulatory membrane components, in eukaryotes [50]; additionally several PI-PI-PLCs are known to be activated via interaction with their pleckstrin homology domains and the βγ subunits of G-proteins [51,52,53]. In mammalian cells, PI-PLC activity is necessary to regulate the formation of transport carrier fission in the trans-Golgi network [54].

As coccoliths are produced in CV, which should be derived from the Golgi body in *E. huxleyi* cells, the intracellular calcification process must have a complex interaction with the endomembrane systems [7,8,10,55,56,57]. Based on this evidence, through endomembrane systems such as the endoplasmic reticulum (ER), the trans-Golgi trafficking can be regulated by IP_3_/Ca^2+^ signaling system via PI-PLC in actively calcifying *E. huxleyi* cells.

The inhibition of coccolith production by the PI-PLC inhibitor U73122 (shown in Figure 4) demonstrates the association of PI-PLC with calcium-induced biomineralization in *E. huxleyi*. The coccolithophore *E. huxleyi* cells release the coccoliths from the cell surface into the medium during culture as revealed by a previous report, which estimated that 10–20 coccoliths per cell were usually detached from exponentially growing cells per day [8]. Our data evaluating the population of coccoliths covering cells and those detached/released into the medium showed that the number of both cell-attached and detached coccoliths, namely the entirety of coccoliths produced, were increased by [Ca^2+^] enrichment, and all coccolith production was hindered by the PI-PLC inhibitor U73122 (Figure 4). These data indicate that PI-PLC must be involved in the intracellular calcification process resulting in coccolith production.

Recently, successful genetic transformation methods have been established for other haptophytes, especially in the coccolithophore *Pleurochrysis carterae* [58,59]. Although *P. carterae* has a calcification mechanism in the subcellular system for intracellular calcification that differs morphologically and physiologically, the basic molecular system of coccolith production, such as calcium accumulation and bicarbonate transport, may be roughly similar. In *E. huxleyi*, the calcium/phosphate-rich phase in a different compartment from the coccolith-producing vesicles functions as a calcium reservoir [60]. Additionally, it has been shown lately that *P. carterae* also contains a calcium storage compartment [61]. Based on this evidence, our finding in this study carefully suggests that PI-PLC functions to accumulate calcium in this reservoir and supports the stimulation of intracellular calcification that is promoted under calcium-rich conditions in *E. huxleyi* cells. Further analysis using such genetic manipulation should be introduced to understand the regulatory mechanism of individual genes related to calcium-induced biomineralization in vivo, although the investigated genetic transformation techniques need to be established in *E. huxleyi*.

## 5. Conclusions

Here, we demonstrate that the biomineralization of *E. huxleyi* CCMP 371 is not entirely dependent on phosphate limitation, which is known to be a primary trigger for coccolith production in other strains of *E. huxleyi*, and the regulatory mechanism appears to differ among strains as they were isolated from various environmental conditions and habitats. Actually, this study clearly showed that the elevation of calcium concentration from 0.1 to 10 mM triggers and promotes the mineralization process of the *E. huxleyi* CCMP 371 strain even under phosphate-sufficient conditions in which coccolith production is usually suppressed in other strains. Based on the RNA-seq and PI-PLC inhibitor experiments, the present study suggests the involvement of PI-PLC in the calcium-induced coccolith formation mechanism in *E. huxleyi* CCMP 371.

## Figures and Tables

**Table 1:** The enriched domains of upregulated DEGs in the PFAM database.

Term ID	Description	Count in Gene Set	FDR
PF14765	Polyketide synthase dehydratase	5 of 18	1.28 × 10^−7^
PF00550	Phosphopantetheine attachment site	5 of 27	3.78 × 10^−7^
PF00975	Thioesterase domain	4 of 19	5.50 × 10^−6^
PF02801	Beta-ketoacyl synthase, C-terminal domain	4 of 19	5.50 × 10^−6^
PF00109	Beta-ketoacyl synthase, N-terminal domain	4 of 24	7.57 × 10^−6^
PF08659	KR domain	5 of 127	0.00016
PF00127	Copper binding proteins, plastocyanin/azurin family	2 of 9	0.0034
PF00063	Myosin head (motor domain)	3 of 57	0.0036
PF08811	Protein of unknown function (DUF1800)	2 of 10	0.0036
PF00388	Phosphatidylinositol-specific phospholipase C, X domain	2 of 19	0.009
PF00169	PH domain	2 of 35	0.0255

**Table 2:** The enriched domains of upregulated DEGs in the Interpro database.

Term ID	Description	Count in Gene Set	FDR
IPR006162	Phosphopantetheine attachment site	5 of 19	2.35 × 10^−7^
IPR013968	Polyketide synthase, ketoreductase domain	5 of 23	2.35 × 10^−7^
IPR020806	Polyketide synthase, phosphopantetheine-binding domain	5 of 19	2.35 × 10^−7^
IPR020807	Polyketide synthase, dehydratase domain	5 of 18	2.35 × 10^−7^
IPR009081	Phosphopantetheine binding ACP domain	5 of 27	2.78 × 10^−7^
IPR036736	ACP-like superfamily	5 of 28	2.78 × 10^−7^
IPR020843	Polyketide synthase, enoylreductase domain	5 of 34	5.60 × 10^−7^
IPR001031	Thioesterase	4 of 13	1.03 × 10^−6^
IPR016039	Thiolase-like	5 of 40	1.03 × 10^−6^
IPR014031	Beta-ketoacyl synthase, C-terminal	4 of 19	3.03 × 10^−6^
IPR020841	Polyketide synthase, beta-ketoacyl synthase domain	4 of 19	3.03 × 10^−6^
IPR014030	Beta-ketoacyl synthase, N-terminal	4 of 21	3.59 × 10^−6^
IPR011032	GroES-like superfamily	5 of 59	3.83 × 10^−6^
IPR008972	Cupredoxin	3 of 33	0.00083
IPR016040	NAD(P)-binding domain	5 of 211	0.0013
IPR018201	Beta-ketoacyl synthase, active site	2 of 6	0.0014
IPR000923	Blue (type 1) copper domain	2 of 8	0.0021
IPR013217	Methyltransferase type 12	2 of 8	0.0021
IPR001609	Myosin head, motor domain	3 of 53	0.0023
IPR014917	Protein of unknown function DUF1800	2 of 10	0.0026
IPR036774	ERV/ALR sulfhydryl oxidase domain superfamily	2 of 12	0.0035
IPR000909	Phosphatidylinositol-specific phospholipase C, X domain	2 of 19	0.0075
IPR001711	Phospholipase C, phosphatidylinositol-specific, Y domain	2 of 23	0.0103
IPR008979	Galactose-binding-like domain superfamily	3 of 103	0.0117
IPR001192	Phosphoinositide phospholipase C family	2 of 27	0.0127
IPR017946	PLC-like phosphodiesterase, TIM beta/alpha-barrel domain superfamily	2 of 30	0.0146
IPR036961	Kinesin motor domain superfamily	3 of 115	0.0146
IPR036291	NAD(P)-binding domain superfamily	5 of 471	0.0234
IPR020845	AMP-binding, conserved site	2 of 44	0.0273
IPR029058	Alpha/Beta hydrolase fold	5 of 527	0.0347
IPR016035	Acyl transferase/acyl hydrolase/lysophospholipase	2 of 59	0.0442

## Supplementary Materials

The following are available online at https://www.mdpi.com/2076-2607/8/9/1389/s1, Figure S1: Analysis of changes in total organic and inorganic carbon (TOC and TIC, respectively) after transferring cells grown in low [Ca^2+^] to 10 mM [Ca^2+^] conditions. (a) Change in TOC concentration in the cell fraction containing cells and coccoliths (cell C/mL). (b) TOC content per cell (cell C/cell). (c) Changes in TIC in the cells (TIC cell), TIC in the culture medium without cells (TIC medium), and TIC in the cell suspension (Total TIC), which should equal the sum of TIC cell and TIC medium, Figure S2: Growth curves of *E. huxleyi* CCMP 371 in the experiments involving treatment with the PI-PLC inhibitor U73122. The cells were cultivated at a calcium concentration ([Ca^2+^]) of 0.1 mM for 1 day (negative control). Then, 10 mM [Ca^2+^] (final concentration) was added without (positive control) and with 0.01, 0.1, and 0.2 μM U73122 (expressed as Ca^2+^ 10 mM + PI-PLC inhibitor 0.01, 0.1, and 0.2, respectively). Cells were harvested after 72 h of treatment (on Day 4) for further flow cytometry analysis, Figure S3: The protein–protein interaction network of differentially expressed genes (DEGs) that were upregulated with coccolith production triggered by the increase in [Ca^2+^]. (a) Phosphoinositide (PI) system. (b) Polyketide synthases (PKSs). (c) Ribonucleoprotein network. Different-colored lines link the nodes and represent the evidence used to predict the interactions (green: neighborhood, black: co-expression, pink: experimental, light violet: protein homology, pale green: text mining), Figure S4: Effect of U73343, analog of U73122 with an inactive effect on PI-PLC activity as a negative control, on calcium-induced coccolith production analyzed using a flow cytometer in *E. huxleyi* CCMP 371. Cells were grown in 0.1 mM [Ca^2+^] (- Control) and transferred to 10 mM [Ca^2+^] (on Day 1) and incubated them for 72 h without (+ Control) and with 0.01, 0.1, and 0.2 μM U73343 (as in Figure 4). (a) Data of forward scatter (FSC) vs. side scatter (SSC) (top), and FSC vs. calcein-positive populations estimated from the coccolith-gated population (middle) and cells (chlorophyll-positive, Chl+) (bottom). (b) Merged histograms of counts vs. calcein-positive populations in cells (Chl+) (upper) and coccoliths (lower). Boxes on the left represent data for the control without inhibitor (pink: 10 mM [Ca^2+^]-treated cells; blue: cells grown in 0.1 mM [Ca^2+^], boxes on the left). Other boxes represent data for samples treated with 0.01, 0.1, and 0.2 μM U733343 (designated as 10 mM Ca^2+^ + U73343 μM) (blue) and 10 mM [Ca^2+^]-treated cells (pink), as indicated in each box. (c) The geometric mean fluorescence intensity (GMFI) of the cells and coccoliths, Table S1: Raw data statistics of RNA sequencing, Table S2: Primers used for qPCR, Table S3: Differentially expressed genes in calcium-induced biomineralization.
