# Supplementary figures and images for: Association of Phosphatidylinositol-Specific Phospholipase C with Calcium-Induced Biomineralization in the Coccolithophore Emiliania huxleyi
Source: Microorganisms. 2020 Sep 10;8(9):1389. doi: 10.3390/microorganisms8091389 (PMC7563939; doi:10.3390/microorganisms8091389)

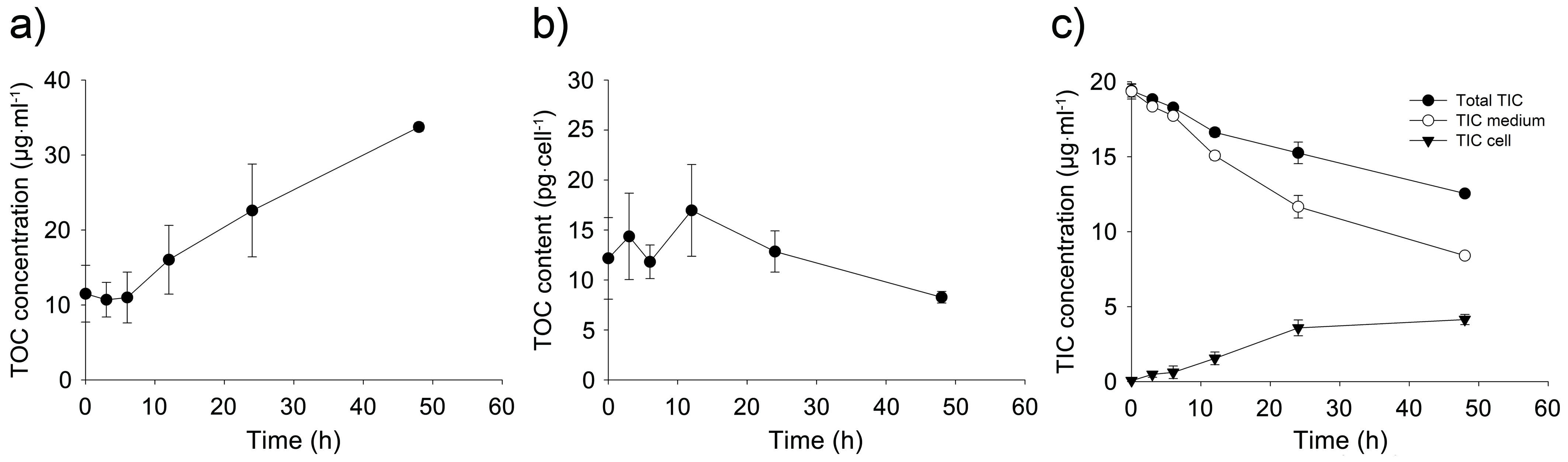

Supplement: Supplementary file 1 [file microorganisms-08-01389-s001.zip › Figure S1.tif]

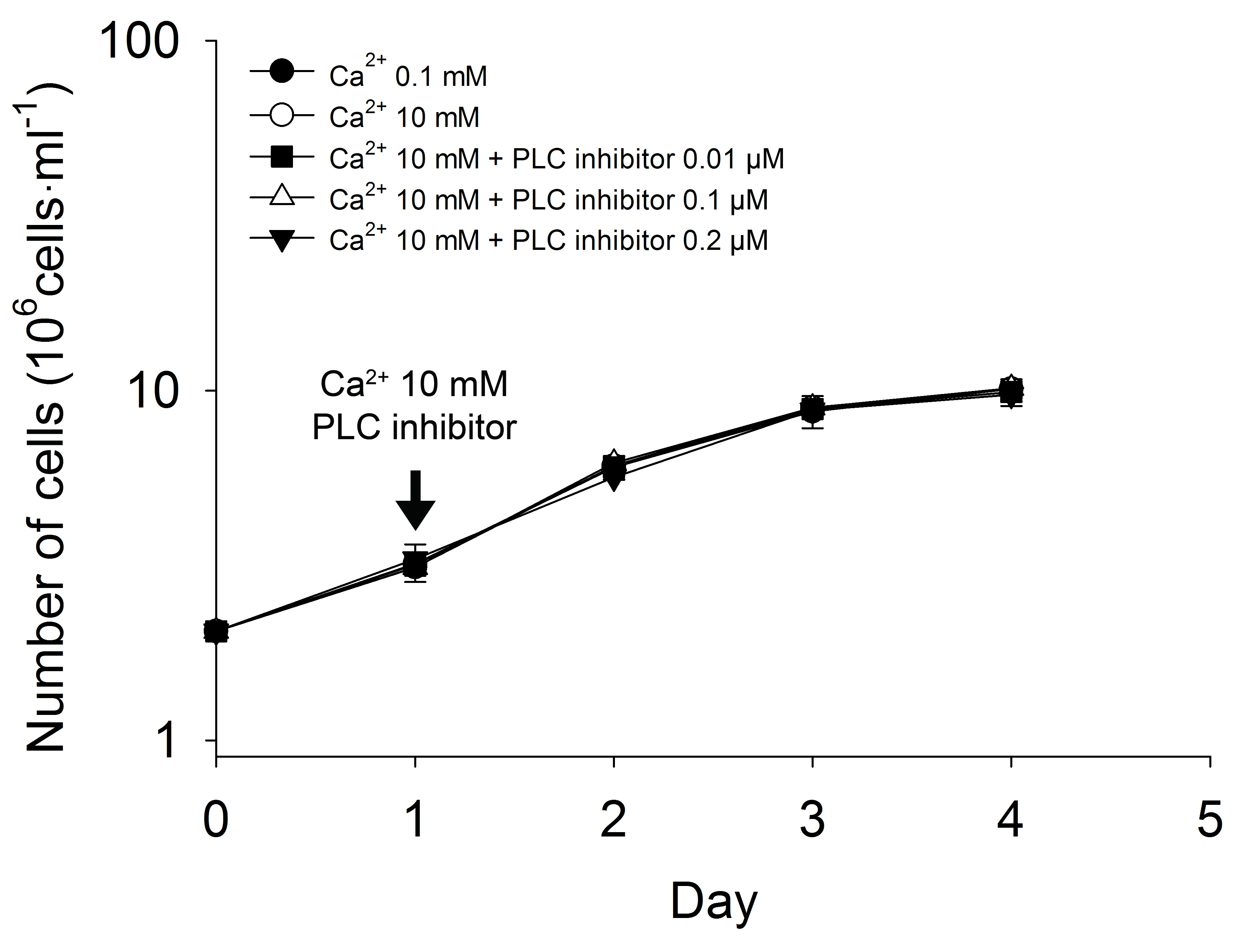

Supplement: Supplementary file 1 [file microorganisms-08-01389-s001.zip › Figure S2.tif]

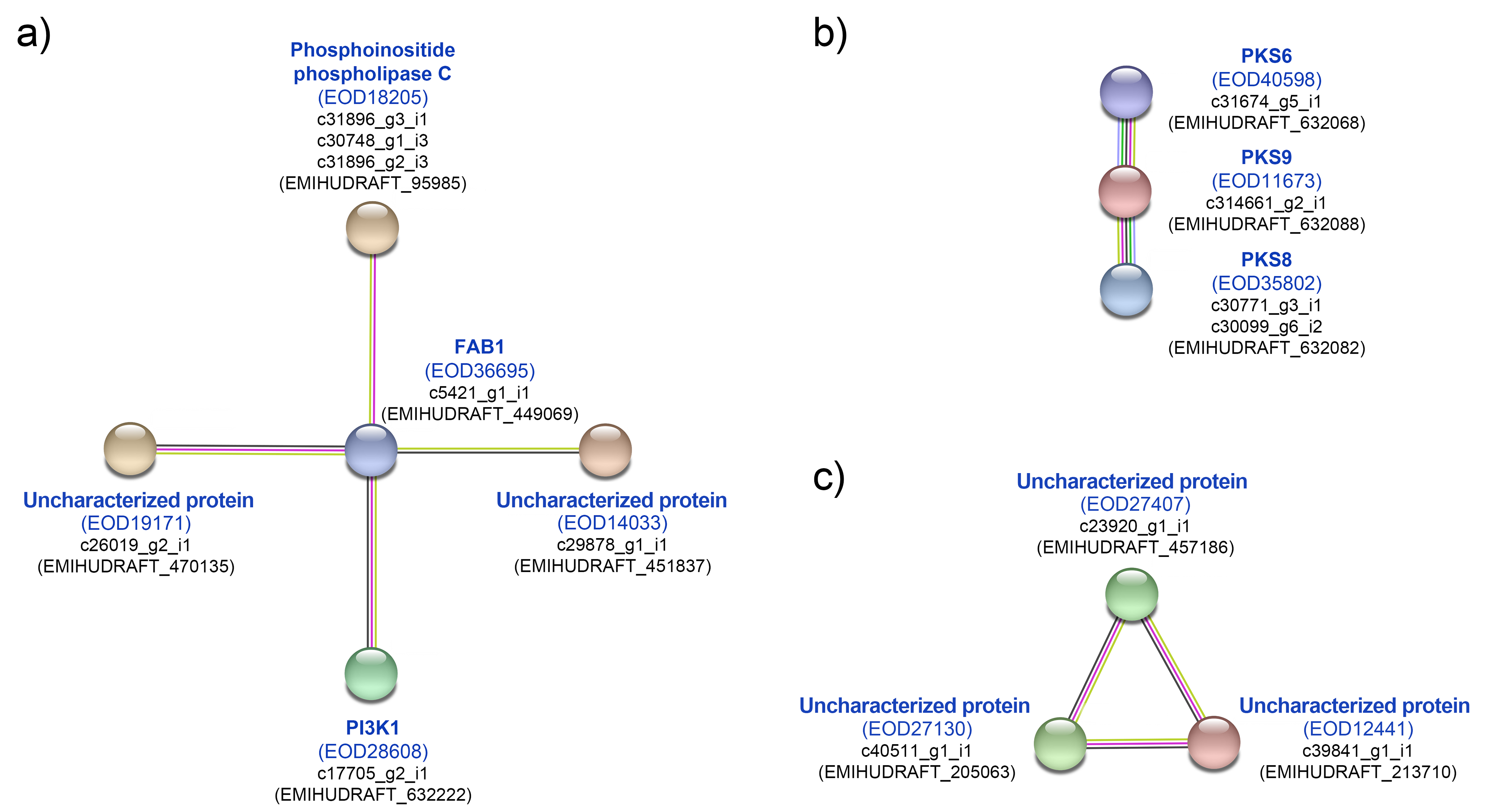

Supplement: Supplementary file 1 [file microorganisms-08-01389-s001.zip › Figure S3.tif]

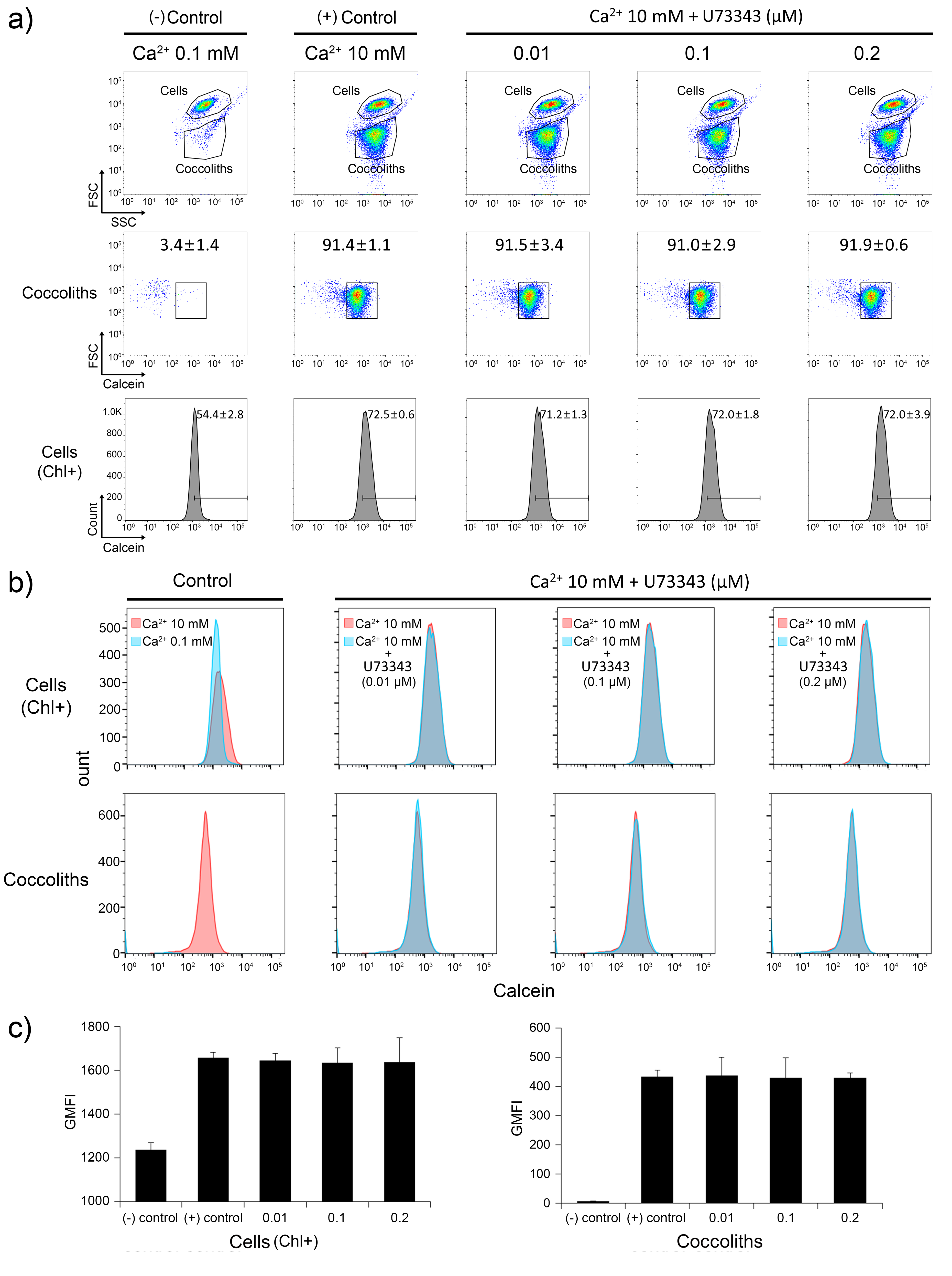

Supplement: Supplementary file 1 [file microorganisms-08-01389-s001.zip › Figure S4.tif]
